# Replacement of Supplemental Fish Oil by Linseed or Soybean Oil Reshapes Hepatic Lipid Metabolism Without Compromising Growth in Juvenile Chinese Soft-Shelled Turtle (*Pelodiscus sinensis*)

**DOI:** 10.3390/ani16132042

**Published:** 2026-07-02

**Authors:** Rui Li, Yilei Guo, Enhao Zhao, Chutian Ge, Jie Sun

**Affiliations:** 1 College of Biological and Environmental Sciences, Zhejiang Wanli University, Ningbo 315100, China; 2023881032@zwu.edu.cn (R.L.); 2024881027@zwu.edu.cn (Y.G.); 2 Institute of Animal Sex and Development, Zhejiang Wanli University, Ningbo 315100, China; m230100420@st.shou.edu.cn; 3 College of Fisheries and Life Sciences, Shanghai Ocean University, Shanghai 201306, China

**Keywords:** *Pelodiscus sinensis*, aquafeed, fish oil replacement, hepatic lipid metabolism, transcriptome

## Abstract

Reducing reliance on supplemental fish oil is central to sustainable aquaculture, but the molecular consequences of replacing it with vegetable oils remain poorly characterized in the juvenile Chinese soft-shelled turtle (*Pelodiscus sinensis*). We evaluated whether full substitution of the supplemental dietary fish oil (FO) with linseed oil (LO) or soybean oil (SO) compromises hepatic lipid metabolism in *Pelodiscus sinensis*. Three isonitrogenous and isolipidic diets, sharing identical fish meal and other ingredient bases and differing only in the supplemental lipid (4% FO, LO or SO), were fed to triplicate groups of juvenile turtles (initial body weight 55.0 ± 0.05 g) for 8 weeks. Growth performance, survival, feed conversion ratio, and serum biochemistry were unaffected. However, both vegetable oil diets altered tissue fatty acid composition, raising n-6 PUFA and lowering n-3 LC-PUFA and the n-3/n-6 ratio in liver and muscle (muscle EPA and DHA each decreased by approximately 40%); the SO group additionally exhibited elevated hepatic malondialdehyde, whereas hepatic lipid droplet area and lipid content did not differ significantly among groups. Liver transcriptomic profiling identified 262 (LO vs. FO) and 214 (SO vs. FO) differentially expressed genes, converging on lipid storage and bile acid metabolism. RT-qPCR confirmed the up-regulation of *PLIN3*, *G0S2* and *APOF* and the down-regulation of *CYP7A1*. Over 8 weeks, replacement of supplemental FO maintained growth without overt impairment while altering tissue fatty acid profiles and the hepatic expression of key lipid metabolism genes.

## Simple Summary

Fish oil is a key ingredient in feeds used in aquaculture, the farming of aquatic animals, because it provides essential fats needed for animal growth and health. However, the global supply of fish oil is limited and increasingly expensive, so reducing the amount of added fish oil in feeds is important for the long-term sustainability of the aquaculture industry. The Chinese soft-shelled turtle is a freshwater reptile widely farmed in China, but it remains unclear how its liver responds when the added fish oil in its feed is replaced by vegetable oils. In this study, we fed juvenile turtles three diets for eight weeks. In the control diet, the added oil was fish oil, while in the other two diets it was fully replaced by linseed oil or soybean oil. The turtles grew equally well on all three diets, but the types of fat stored in their tissues were substantially altered, and the liver lipid content showed no significant change. By examining liver gene activity, we found that several genes controlling how fat is stored and broken down were notably changed. These findings provide useful information on how this species responds to vegetable-oil-based feeds.

## 1. Introduction

Long-chain polyunsaturated fatty acids (LC-PUFA), particularly eicosapentaenoic acid (EPA) and docosahexaenoic acid (DHA), play essential roles in supporting growth, neural development, immune competence, and metabolic regulation in farmed aquatic animals [1]. In aquafeeds, these biologically important fatty acids are predominantly supplied through fish oil (FO) [2]. In 2021, the aquaculture sector consumed approximately 0.74 million tons of FO, representing nearly 74% of global FO production [3]. Rapid growth in aquaculture, coupled with limited marine oil resources, has tightened the balance between FO supply and demand, making the identification of sustainable lipid alternatives a long-standing priority for the aquaculture industry [4].

A variety of vegetable oils derived from linseed, soybean, palm, rapeseed [5], and sunflower oil [6] have been studied as partial or complete replacements for FO in the diets of various aquatic species [7]. Despite differences among species and oil types, a consistent outcome of FO replacement is a decline in tissue n-3 LC-PUFA and a lowered n-3/n-6 PUFA ratio, reflecting the absence of preformed EPA and DHA in vegetable oils; in contrast, the consequences for growth and hepatic lipid handling are far more variable. For example, complete replacement of FO with LO did not impair growth in the large yellow croaker (*Larimichthys crocea*), yet still shifted tissue profiles toward higher C18 PUFA and lower n-3 LC-PUFA [8]; in rainbow trout (*Oncorhynchus mykiss*), high vegetable oil inclusion lowered muscle EPA and DHA and the n-3/n-6 ratio [9]; and in Nile tilapia (*Oreochromis niloticus*), FO replacement additionally increased hepatic lipid deposition and altered the expression of lipid metabolism genes [10]. Thus, while the fatty acid response to FO replacement is broadly predictable, its impact on growth and hepatic lipid metabolism appears strongly species-dependent, underscoring the need for species-specific evaluation.

Among vegetable lipid sources, LO and SO have received particular attention because of their contrasting fatty acid compositions. LO is rich in α-linolenic acid (ALA, 18:3n-3), while SO is rich in linoleic acid (LA, 18:2n-6). As the principal C18 precursors of the n-3 and n-6 PUFA families, respectively, ALA and LA make LO and SO representative models for contrasting “n-3-type” and “n-6-type” vegetable oils; comparing them therefore allows the effects of precursor type, rather than merely the removal of preformed LC-PUFA, to be examined. Other candidate oils are less suitable for this purpose: palm oil is dominated by saturated fatty acids and is low in C18 PUFA, while rapeseed and sunflower oils contain mixed or predominantly n-6 profiles that do not provide a clear n-3 versus n-6 contrast [11]. LO and SO also have the practical advantages of wide availability and established use in aquafeeds. Freshwater fish generally possess some ability to convert C18 PUFA into LC-PUFA [12], positioning LO and SO as promising alternatives to sustain LC-PUFA biosynthesis in aquafeeds. Nonetheless, the appropriate level of FO replacement varies according to oil type, species, and developmental stage, and excessive substitution may negatively affect physiological performance and product quality [13]. Compared with partial substitution, complete replacement of FO presents greater nutritional challenges, primarily due to reductions in dietary n-3 LC-PUFA and imbalances in n-3/n-6 fatty acid ratios [14]. Previous research has shown that fully replacing FO can influence multiple physiological processes in aquatic animals in a species- and dose-dependent manner [15]. Specifically, complete replacement has been associated with reduced growth performance [16,17], altered antioxidant capacity and elevated oxidative stress [18,19], along with alterations in lipid deposition and pathways related to lipid metabolism [20].

The Chinese soft-shelled turtle (*Pelodiscus sinensis*) is an economically important freshwater reptile widely cultured in China, valued for its favorable nutritional profile, tender flesh, and high consumer acceptance. As production intensity and market demand have continued to grow, optimizing dietary lipid sources to reduce reliance on FO has become an increasingly relevant goal for the sustainable development of this industry. However, mechanistic studies of FO replacement have been conducted largely in teleost fish [21,22], whereas comparable evidence in cultured reptiles remains scarce. As a reptile, *P. sinensis* may differ from teleosts in hepatic lipid handling, yet the few available studies in this species have addressed mainly growth performance and tissue fatty acid composition [23,24], and how supplemental FO replacement affects hepatic lipid metabolism at the transcriptional level remains essentially unknown. This gap is the focus of the present study.

Combining transcriptomic profiling with targeted gene expression analysis can help clarify how dietary lipid sources affect hepatic lipid handling beyond what is captured by phenotypic measurements alone [25]. We therefore hypothesized that full replacement of the supplemental FO with LO or SO would not impair the growth of juvenile *P. sinensis*, but would alter tissue fatty acid composition and influence the expression of genes involved in the hepatic lipid metabolism, with the n-3-rich (LO) and n-6-rich (SO) oils exerting partly distinct effects. With this in mind, the present study used juvenile *P. sinensis* fed diets in which the supplemental FO was fully replaced with either LO or SO. We examined growth performance, tissue fatty acid composition, serum biochemistry, hepatic antioxidant indices, liver histology, hepatic transcriptome profiles, and RT-qPCR validation of selected lipid metabolism genes, in order to obtain a more integrated view of how this dietary substitution influences both the physiology and the hepatic gene expression of this species. The findings are expected to provide information useful for the further evaluation of vegetable-oil-based diets for *P. sinensis*.

## 2. Materials and Methods

### 2.1. Animal Ethics

All animal experimentation protocols were approved by the Institutional Animal Care and Use Committee at Zhejiang Wanli University (Approval No. 20240918001), and complied with the Regulations on the Management of Laboratory Animals in China.

### 2.2. Diets Formulation

Three experimental diets were formulated to contain about 45.0% crude protein and 7.8% crude lipid (Table 1). White fish meal (52%), poultry by-product meal (6%) and soybean protein concentrate (10%) constituted the primary protein sources across all diets. The FO diet served as the control; in the other two diets, the supplemental FO was fully replaced with LO or SO, respectively. It should be noted that white fish meal (52% of diet) inherently contributed approximately 3.1% crude lipid to all three diets, equivalent to 39% of total dietary lipid; the dietary substitution therefore applies specifically to the 4% supplemental lipid fraction rather than to the entire pool of fish-derived lipid in the formulation.

### 2.3. Experimental Turtles and Culture Conditions

We conducted the experiment at an aquaculture facility in Ningbo (Zhejiang province, China), using cement pools (3.2 m × 2.5 m × 1.1 m) corresponding to an initial stocking density of 3.75 ind m^−2^ (initial biomass density 0.21 kg m^−2^) which were disinfected with chlorine dioxide prior to use. The experimental juvenile turtles were purchased from Yuyao Lengjiang Chinese soft-shelled turtle Industry Co., Ltd. (Yuyao, China). Prior to the formal trial, the turtles underwent a one-week acclimation period in the experimental pools, during which they were fed the control diet. Before each feeding, the powdered diet was mixed with water at a ratio of 1:0.75 (*w*/*w*) to produce a dough and then fixed onto a wooden feeding platform. After acclimation, all *P. sinensis* were fasted for 24 h. Three dietary treatments were each randomly assigned to triplicate cement pools, with each pool stocked with 30 healthy turtles of comparable body mass (55.0 ± 0.05 g). These juvenile turtles were approximately 10 months old at the start of the trial. The turtles were fed twice a day at 6:00 and 17:00, and mortality was recorded in order to calculate survival rate (SR) over an 8-week period. Daily feed amounts were set at 1–3% of the turtles’ body weight and adjusted every two weeks according to the weight of ten randomly selected turtles. To determine the feed conversion ratio (FCR), unused feed was collected, dried, and weighed. A principle of maximizing natural conditions guided the rearing environment management throughout the feeding trial. The experiment was performed outdoors with naturally fluctuating seasonal water temperature. All tanks for the three dietary treatments were arranged in the same culture area with a unified water supply and were reared simultaneously; the water temperature of each tank was recorded daily, and no obvious temperature divergence among tanks was observed during the feeding trial. Specific parameters were monitored throughout the trial and maintained as follows: water temperature, 21.2–29.6 °C; dissolved oxygen, above 5.5 mg/L; ammonia nitrogen, below 0.5 mg/L; and pH, 6.5–8.0.

### 2.4. Sample Collection

After the 8-week feeding trial, all turtles were fasted for 24 h prior to sampling. Data on turtle numbers and body weights in each pool were recorded for calculating SR, weight gain rate (WGR), specific growth rate (SGR) and FCR. Three turtles were randomly chosen and anesthetized using MS-222 (Sigma-Aldrich, St. Louis, MO, USA, 97% purity) at a 1:10,000 dilution. Arterial blood was extracted via the carotid artery with a 1 mL syringe, followed by serum separation through centrifugation (4000× *g*, 12 min) for biochemical analysis. Concurrently, muscle and liver samples were taken for compositional analysis, then kept at −80 °C after labeling. The visceral mass and liver weight of these three turtles were also recorded for the determination of the viscerosomatic index (VSI) and hepatosomatic index (HSI). For each treatment, nine juvenile turtles were sampled, comprising three turtles from each of the three replicate pools. Within each pool, total RNA was extracted individually from the three livers and then pooled in equal amounts to form one biological replicate; the three pools per treatment thus provided three biological replicates (*n* = 3), with the pool serving as the experimental unit. The liver tissue from each replicate was then allocated to three portions for different analyses. One portion was fixed in 4% paraformaldehyde (PFA) for H&E staining, and another was frozen directly in liquid nitrogen and held at −80 °C for Oil Red O staining. The remaining portion, intended for gene expression and transcriptomic analyses, was rinsed with phosphate-buffered saline, placed in a 2.0 mL RNase-free cryogenic tube, snap-frozen in liquid nitrogen, and kept at −80 °C.

### 2.5. Proximate Composition Analysis

The proximate composition of the experimental diets, liver and muscle was analyzed following the standard procedures of the Association of Official Analytical Chemists (AOAC, 16th edition) [27]. The experimental diets were vacuum freeze-dried to constant weight prior to analysis, whereas liver and muscle were analyzed as fresh tissue. Crude protein was quantified as total nitrogen × 6.25 using an automated Kjeldahl analyzer (Kjeltec™ 8200, FOSS Analytical, Hillerød, Denmark), while crude lipid was measured by Soxhlet extraction on a fat extraction system (Soxtec 2050, FOSS Analytical, Hillerød, Denmark). Each diet was analyzed in duplicate and each tissue sample in triplicate (*n* = 3 pooled biological replicates per group).

### 2.6. Fatty Acid Composition Analysis

Fatty acid composition of the experimental diets and of liver and muscle samples was determined by gas chromatography–mass spectrometry, with three replicate measurements per sample. Approximately 100 mg of freeze-dried, finely ground material was weighed into a stoppered glass tube and saponified with 3 mL of 1 mol/L KOH-methanol in a 75–80 °C water bath for 20 min. After cooling, the fatty acids were methylated with 3 mL of 2 mol/L HCl-methanol under the same conditions (75–80 °C, 20 min). The resulting fatty acid methyl esters (FAMEs) were extracted by vortexing with 1 mL of chromatography-grade n-hexane and a small volume of ultrapure water. Once the phases had separated, the upper n-hexane layer was centrifuged (4000× *g*, 5 min), and the supernatant was transferred to a vial and held at −20 °C in the dark until analysis.

FAMEs were separated on an Agilent 8890 GC-MS system (Agilent Technologies, Santa Clara, CA, USA) fitted with an HP-5MS capillary column (30 m × 0.25 mm × 0.25 µm), using helium as the carrier gas at a split ratio of 50:1 and an injection volume of 1 µL. The injector and transfer line were held at 250 °C and 260 °C, respectively. The oven was programmed from 150 °C (2 min), raised to 200 °C at 10 °C/min, and then to 240 °C at 5 °C/min (held 15 min). Individual FAMEs were identified against a Supelco 37-component FAME mix standard (CRM47885, Merck, Darmstadt, Germany) and quantified by peak area normalization, with each fatty acid expressed as a percentage of total identified fatty acids. Dietary fatty acid profiles are given in Table 2.

### 2.7. Serum Biochemistry Parameters Analysis

Serum biochemistry was assessed using commercial kits (Nanjing Jiancheng Bioengineering Institute, Nanjing, China) according to the manufacturer’s protocols. The assays covered triglyceride (TG) and total cholesterol (T-CHO) as general lipid indices, the two lipoprotein cholesterol fractions (LDL-C and HDL-C), the activities of alanine aminotransferase (ALT) and aspartate aminotransferase (AST) as indicators of hepatic function.

### 2.8. Hepatic Antioxidant Parameters

Weighed liver samples were homogenized in nine volumes of chilled normal saline (1:9, *w*/*v*) in an ice-water bath. The homogenates were centrifuged at 4000× *g* for 12 min, and the supernatants were collected for subsequent analyses. Using commercial kits (Nanjing Jiancheng Bioengineering Institute, Nanjing, China) and following the supplier’s guidelines, the assays covered total antioxidant capacity (T-AOC) as a measure of overall antioxidant status, malondialdehyde (MDA) as an indicator of lipid peroxidation, and the activities of two antioxidant enzymes, superoxide dismutase (SOD) and catalase (CAT).

### 2.9. Histochemical and Histological Analysis

Hepatic lipid distribution was visualized by Oil Red O staining. Snap-frozen liver samples were embedded in Tissue-Tek O.C.T. Compound (Sakura Finetek USA, Torrance, CA, USA) and cut into 10-μm cryosections (CryStar NX50, Thermo Fisher Scientific, Waltham, MA, USA), with the tissue kept frozen throughout to avoid thawing. After brief fixation, sections were stained with a modified Oil Red O kit (Solarbio, Beijing, China) following the kit instructions. Staining, image capture and quantification followed Mehlem et al. [28], with minor modifications. Three sections were prepared from the liver tip of each of three animals per group (*n* = 3 per group), and ten non-overlapping fields per section were systematically sampled across the entire section and photographed at ×200 magnification; fields showing tissue rupture or folding were excluded. All slides were coded to mask group identity, and image acquisition and analysis were performed by two independent researchers blinded to group assignment. The percentage of Oil Red O-positive area in each image was quantified using ImageJ (v.1.54p99, National Institutes of Health, Bethesda, MD, USA) software.

Liver histology was further examined by hematoxylin and eosin (H&E) staining. Tissues fixed in 4% paraformaldehyde for 24 h were processed and paraffin-embedded as described by Huang et al. [29], sectioned at 6 μm, and stained with H&E. Three sections were prepared from the liver tip of each of three animals per group (*n* = 3 per group), and ten non-overlapping fields per section were systematically examined and photographed at ×200 magnification; fields showing tissue rupture or folding were excluded. All slides were coded to mask group identity, and sections were assessed by two independent researchers blinded to group assignment. Both histochemical and histological sections were examined and imaged using an upright optical microscope (Nikon, Tokyo, Japan).

### 2.10. RNA Extraction and cDNA Synthesis

Total RNA was isolated individually from each of the three liver tissues with TRIzol reagent (Life Technologies, Carlsbad, CA, USA) following the manufacturer’s protocol with minor modifications. Briefly, approximately 100 mg of liver tissue was homogenized in 1 mL of TRIzol with a tissue grinder (60 Hz, 2 min) and kept on ice; after addition of 200 µL of chloroform and incubation on ice for 15 min, the phases were separated by centrifugation (12,000× *g*, 15 min, 4 °C) and the upper aqueous phase was collected. RNA was precipitated with an equal volume of pre-chilled isopropanol (15 min on ice; 12,000× *g*, 15 min, 4 °C), washed twice with 75% ethanol prepared in DEPC-treated water (7500× *g*, 5 min), air-dried, and dissolved in 20 µL of RNase-free water. RNA concentration and purity were checked on a NanoDrop One spectrophotometer (Thermo Fisher Scientific, Waltham, MA, USA), and integrity was confirmed by 1% agarose gel electrophoresis; only samples with an A260/A280 of 1.8–2.0 and an A260/A230 above 2.0 were used further. Equal amounts of RNA from the three individuals within each tank were then pooled in equal quantity to constitute one biological replicate (*n* = 3 per group), which was used for both RNA sequencing and RT-qPCR. First-strand cDNA was then synthesized from 2 µg of total RNA in a 20 µL reaction using the RevertAid First Strand cDNA Synthesis Kit (Thermo Fisher Scientific, Waltham, MA, USA) with oligo(dT) primers, following the supplier’s protocol. Reactions were incubated at 42 °C for 60 min and terminated at 70 °C for 5 min, and the cDNA was stored at −20 °C until use.

### 2.11. RNA-Seq Library Construction, Sequencing, and Bioinformatic Analysis

Total RNA prepared as described in Section 2.10 was submitted for sequencing. Three pooled biological replicates per dietary group (*n* = 3, each pool comprising livers from three turtles) were submitted to Beijing Novogene Bioinformatics Technology Co., Ltd. (Beijing, China) for library construction and sequencing on an Illumina platform using a 150 bp paired-end (PE150) strategy. Each sample yielded approximately 6 G of raw data, a depth widely used for gene-level expression profiling in non-model aquaculture species with a reference genome and sufficient for reliable transcriptome quantification. All nine libraries were prepared and sequenced in a single batch, so batch effects were not a concern. Prior to library construction, RNA integrity was assessed by the sequencing provider on an Agilent 5400 Fragment Analyzer, with RIN values ranging from 5.9 to 7.4 (Table A1). After quality filtering with fastp (v.1.0.0) [30] to remove adapter-containing reads, reads with more than 10% N bases, and low-quality reads, an average of 47.4 million clean reads (range 44.6–50.2 million) were obtained per sample, with Q30 ≥ 94.3% and a mean GC content of 48.3% (Table A1). Clean reads were aligned to the *P. sinensis* reference genome assembly GCF_000230535.1 using HISAT2 (v.2.0.5) [31], with an average total mapping rate of 84.9% (range 84.4–85.3%) and an average unique mapping rate of 81.3% (range 80.8–81.8%) across all samples (Table A2).

**Figure A1:**
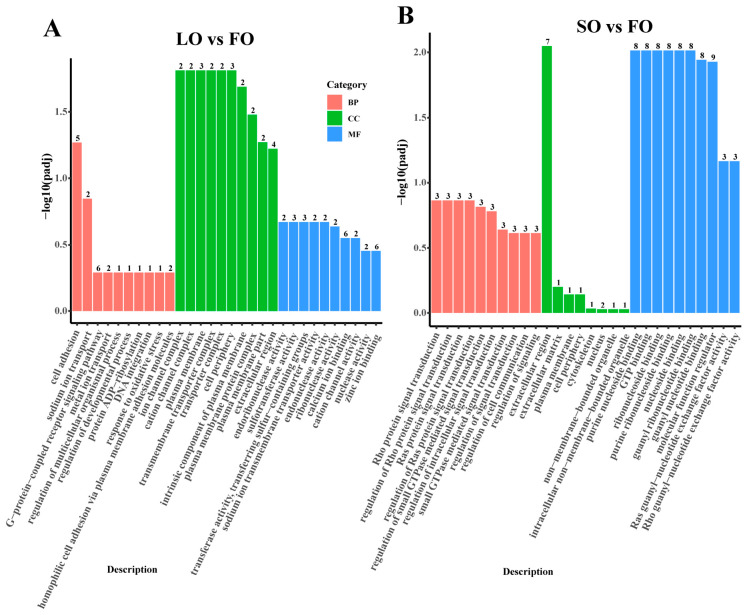
Gene Ontology (GO) enrichment analysis of differentially expressed genes (DEGs) in the (**A**) LO vs. FO and (**B**) SO vs. FO comparisons. The top enriched GO terms are shown for each category: biological process (BP, red), cellular component (CC, green), and molecular function (MF, blue). The *y*-axis represents the enrichment significance expressed as −log_10_(adjusted *p*-value), and the number above each bar indicates the number of DEGs assigned to that term. Adjusted *p*-values were obtained using the Benjamini–Hochberg method.

Gene-level read counts were generated with featureCounts (v.1.5.0) [32] and normalized to fragments per kilobase of transcript per million mapped reads (FPKM). Overall similarity among samples and the presence of potential outliers were examined by principal component analysis, which separated the dietary groups while replicates clustered together, with no outlier samples detected. Differential expression between dietary groups was tested with DESeq2 (v.1.42.0) [33] under a negative binomial model. Genes showing |log2 fold change| ≥ 1 with a Benjamini–Hochberg adjusted *p* < 0.05 were regarded as differentially expressed, and these genes were subjected to KEGG pathway enrichment analysis using the clusterProfiler package (v.4.8.1) [34]. Gene Ontology (GO) enrichment analysis (biological process, cellular component, and molecular function) was performed for the same gene sets with clusterProfiler, with adjusted *p* < 0.05 considered significant (Figure A1). The raw RNA-seq data have been deposited in Zenodo (https://doi.org/10.5281/zenodo.19660547).

### 2.12. Real-Time Quantitative PCR (RT-qPCR)

RT-qPCR was carried out on a CFX96 Touch Real-Time PCR Detection System (Bio-Rad, Hercules, CA, USA) with 2× Universal SYBR Green Fast qPCR Mix (ABclonal Technology, Wuhan, China), using the cDNA prepared as described in Section 2.10. Each 12.5 µL reaction comprised 6.25 µL of SYBR Green Mix, 1 µL of cDNA template, 0.5 µL of each primer (10 µM; 400 nM final), and nuclease-free water. Cycling began with denaturation at 95 °C for 3 min, followed by 40 cycles of 95 °C for 10 s, 58 °C for 30 s and 72 °C for 30 s, and ended with a melt-curve step (65–95 °C, 0.5 °C increments) to verify amplification specificity. No-template controls were included on each plate, and every biological replicate was assayed in three technical replicates.

Primer sequences for all target and reference genes are listed in Table 3. Primer specificity was confirmed by single-peak melt curves and a single band on agarose gels. For each primer pair, amplification efficiency was derived from the slope of a standard curve based on five serial 5-fold dilutions of pooled cDNA; efficiencies ranged from 90.5% to 107.4%, with R^2^ ≥ 0.995 (Table 3).

*GAPDH* was used as the single internal reference gene, in line with previous gene-expression studies in *P. sinensis* [35,36]. Across all liver samples in the present study, the mean Cq value of *GAPDH* was 19.2 with a standard deviation below 0.5 cycles among the three dietary groups, supporting its stable expression under the experimental conditions. Relative expression of target genes was calculated by the 2^−ΔΔ*Cq*^ method [37] with normalization to *GAPDH* and to the mean of the FO group. Each result represents three biological replicates (*n* = 3 pooled samples per dietary group), each measured in triplicate technical replicates.

### 2.13. Calculations and Statistical Methods

Growth performance and morphometric indices were calculated using the following formulas:

Survival rate (SR, %) = N_t_ × 100/N_0_,

Weight gain rate (WGR, %) = (W_t_ − W_0_) × 100/W_0_,

Specific growth rate (SGR, % day^−1^) = (ln W_t_ − ln W_0_) × 100/d,

Feed conversion ratio (FCR) = feed consumed (g)/weight gain (g),

Viscerosomatic index (VSI, %) = visceral weight × 100/body weight,

Hepatosomatic index (HSI, %) = liver weight × 100/body weight,

Where N_0_ and N_t_ represent the initial and final number of turtles, respectively; W_0_ and W_t_ represent the initial and final turtles body weight, and d is the duration of the feeding trial.

All quantitative data are expressed as the mean ± standard error of the mean (mean ± SEM; *n* = 3 pooled biological replicates per group). Prior to analysis, the normality of data within each group was assessed by the Shapiro–Wilk test, and the homogeneity of variances by the Brown–Forsythe test; both assumptions were satisfied. Group differences were evaluated by one-way analysis of variance (ANOVA); when a significant overall effect was detected, Tukey’s honestly significant difference test was applied for pairwise comparisons. Differences were considered statistically significant at *p* < 0.05. The Oil Red O-stained area was quantified with ImageJ, statistical analyses and graphing were carried out in GraphPad Prism 8 (GraphPad Software, San Diego, CA, USA), and the log2FC heatmaps were generated in RStudio (v.4.3.2, Posit Software, Boston, MA, USA) using the pheatmap package (v.1.0.13). Given the limited number of biological replicates (*n* = 3 per group), the statistical power of these tests is acknowledged as a limitation, and the results are interpreted accordingly.

## 3. Results

### 3.1. Growth Performance and Survival

No significant differences were revealed in SR, FBW, WGR, SGR, FCR, VSI, or HSI among juvenile *P. sinensis* subjected to different dietary treatments (Table 4).

### 3.2. Liver and Muscle Proximate Composition and Fatty Acid Composition

Compared with the FO group, dietary replacement of LO or SO did not result in significant changes in crude protein or crude lipid contents in either liver or muscle tissues (Table 5).

In the liver (Table 6), the LO and SO groups showed significantly higher levels of LA and total n-6 PUFA but lower MUFA and DHA than the FO group (*p* < 0.05). ALA was elevated specifically in the LO group, whereas reductions in EPA and ARA were confined to the SO group (*p* < 0.05). SFA was also significantly lower in the SO group than in the FO group (*p* < 0.05). Total n-3 PUFA displayed opposite responses in the two treatments, increasing in the LO group but decreasing in the SO group relative to the FO group (*p* < 0.05). Consequently, the n-3/n-6 PUFA ratio in both the LO and SO groups was significantly reduced (*p* < 0.05).

A comparable pattern was observed in muscle (Table 6): Relative to the FO group, both vegetable oil groups had higher LA and total n-6 PUFA but lower EPA, DHA and total n-3 PUFA, together with a reduced n-3/n-6 PUFA ratio (*p* < 0.05). As in the liver, ALA was elevated only in the LO group (*p* < 0.05). SFA decreased significantly in the SO group (*p* < 0.05), while MUFA remained unchanged across the three groups (*p* > 0.05).

### 3.3. Serum Biochemical Parameters

Serum biochemical parameters did not differ significantly among the FO, LO and SO groups (*p* > 0.05). These parameters included TG, T-CHO, HDL-C, LDL-C, as well as the activities of ALT and AST (Table 7).

### 3.4. Hepatic Antioxidant Capacity and Antioxidant Gene Expression

**Figure 1:**
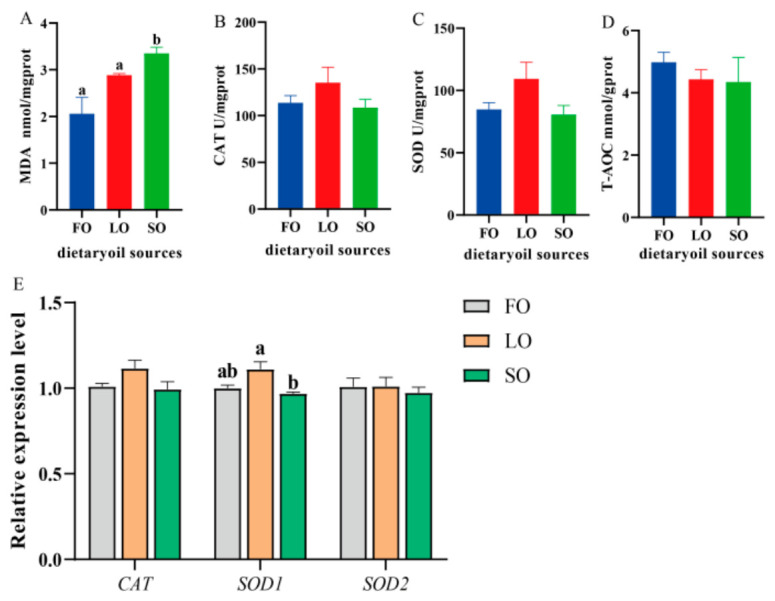
Effects of replacing dietary fish oil with linseed oil or soybean oil on hepatic antioxidant indices and the mRNA expression of antioxidant genes in juvenile *P. sinensis*. (**A**) Malondialdehyde (MDA) content; (**B**) catalase (CAT) activity; (**C**) superoxide dismutase (SOD) activity; (**D**) total antioxidant capacity (T-AOC); (**E**) relative mRNA expression of *CAT*, *SOD1* and *SOD2*. Values are presented as mean ± SEM (*n* = 3 pooled biological replicates per group). Bars labeled with different letters differ significantly (*p* < 0.05, Tukey’s HSD test); the absence of letters indicates no significant difference among groups.

With the exception of a significant increase in MDA in the SO group (*p* < 0.05), the activities of CAT, SOD, and T-AOC remained comparable among all groups (*p* > 0.05) (Figure 1). Similarly, the gene expression levels of *SOD1*, *SOD2* and *CAT* did not differ significantly from the FO group in either the LO or SO groups (*p* > 0.05) (Figure 1).

### 3.5. Histological Analysis in Liver

**Figure 2:**
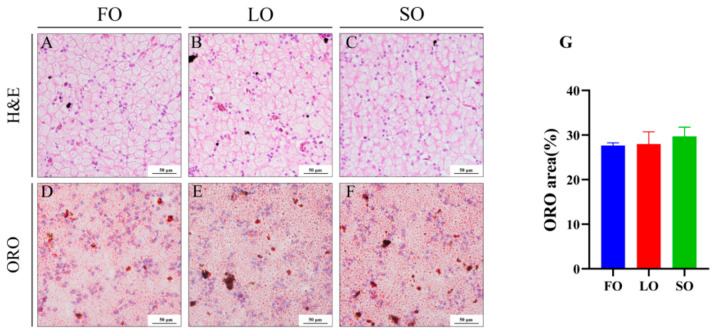
Histological and histochemical analysis of liver tissue from juvenile *P. sinensis* fed FO, LO and SO diets. (**A**–**C**) Representative H&E staining images of the FO, LO and SO groups, respectively. (**D**–**F**) Representative Oil Red O staining images of the FO, LO and SO groups, respectively. (**G**) Quantification of Oil Red O stained area expressed as a percentage of total image area. Values are mean ± SEM (*n* = 3). Scale bars, 50 μm.

Hematoxylin and eosin staining showed that hepatocytes were generally well organized with relatively uniform cytoplasm in all three groups, with only a few scattered cytoplasmic vacuoles and no marked differences in vacuolation among the three groups upon qualitative inspection (Figure 2A–C). Oil Red O staining likewise showed sparse, evenly distributed lipid droplets across the three groups (Figure 2D–F). And image-based quantification confirmed that the Oil Red O-positive area did not differ significantly among groups (*p* > 0.05) (Figure 2G).

### 3.6. T-CHO and TG Levels in Liver and Muscle

**Figure 3:**
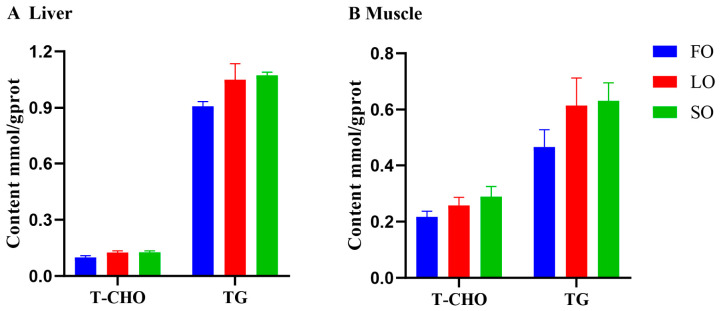
Effects of replacing dietary fish oil with linseed oil or soybean oil on the contents of total cholesterol (T-CHO) and triglyceride (TG) in (**A**) liver and (**B**) muscle of juvenile *P. sinensis*. Values are presented as mean ± SEM (*n* = 3 pooled biological replicates per group). No significant differences are detected among groups (*p* > 0.05, Tukey’s HSD test).

Replacement of FO with LO or SO resulted in a non-significant differences in T-CHO and TG levels in both liver and muscle (*p* > 0.05) (Figure 3).

### 3.7. Liver Transcriptome

**Figure 4:**
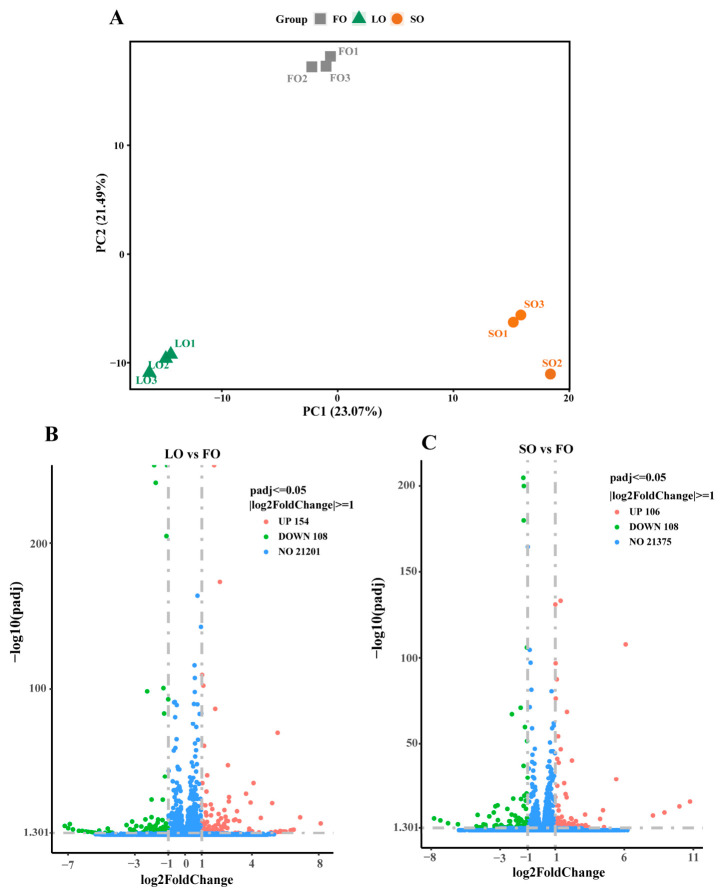
Differential transcriptomic profiles of liver in juvenile *P. sinensis* fed FO, LO and SO diets. (**A**) Principal component analysis of liver transcriptomes (*n* = 3 per group); (**B**,**C**) volcano plots of differentially expressed genes for the LO vs. FO (**B**) and SO vs. FO (**C**) comparisons; up-regulated and down-regulated genes are shown as red and blue dots, respectively. Differentially expressed genes were defined by |log2 fold change| ≥ 1 and adjusted, *p* < 0.05.

Principal component analysis of the liver transcriptomes separated the FO, LO and SO groups clearly, while replicates within each group clustered together (Figure 4A). Differentially expressed genes (DEGs) were defined by |log2 fold change| ≥ 1 together with a Benjamini–Hochberg adjusted *p* < 0.05. On this basis, the LO group differed from the FO group by 262 DEGs (154 up- and 108 down-regulated; Figure 4B), whereas the comparison of the SO and FO groups yielded 214 DEGs (106 up- and 108 down-regulated; Figure 4C).

**Figure 5:**
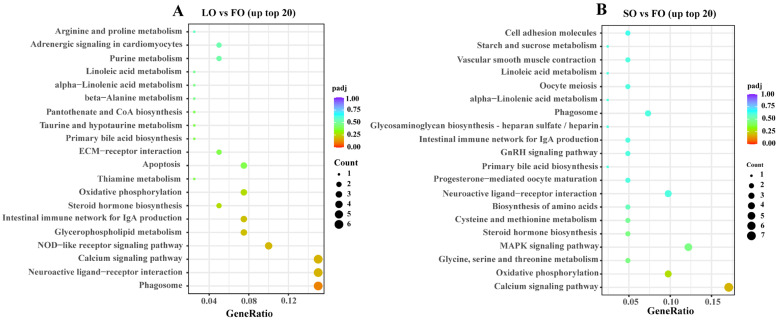
KEGG pathway enrichment analysis of differentially expressed genes in the liver of juvenile *P. sinensis* fed FO, LO and SO diets. (**A**) Top 20 enriched pathways for LO vs. FO comparison; (**B**) top 20 enriched pathways for SO vs. FO comparison. On each plot, the *x*-axis gives the gene ratio and the *y*-axis lists the pathways. Dot color indicates the adjusted *p* value (red, lower; blue, higher), and dot size represents the number of differentially expressed genes mapped to the pathway.

KEGG enrichment analysis did not identify any pathway that reached statistical significance after multiple-testing correction in either the LO vs. FO or SO vs. FO comparison (adjusted *p* > 0.05). Among the top 20 enriched pathways in both comparisons, several were functionally related to lipid and steroid metabolism, including α-linolenic acid metabolism, linoleic acid metabolism, primary bile acid biosynthesis, and steroid hormone biosynthesis (Figure 5). Given their relevance to dietary lipid utilization, these pathways were considered of particular interest and were used to guide the selection of candidate genes for subsequent validation.

**Figure 6:**
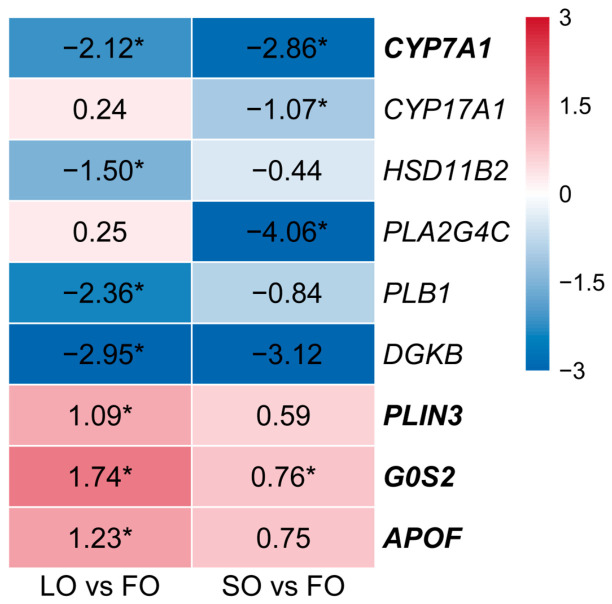
Heatmap of log2 fold changes (log2FC) for nine differentially expressed genes related to hepatic lipid metabolism, in the LO vs. FO and SO vs. FO comparisons. Color indicates the direction and magnitude of log2FC relative to the FO group (red, up-regulation; blue, down-regulation). Asterisks (*) within cells denote statistically significant differential expression (DESeq2, adjusted *p* < 0.05). Gene symbols in bold were independently validated by RT-qPCR (Figure 7); the remaining genes are presented as RNA-seq-derived findings only.

To examine these lipid-related processes in more detail, nine DEGs involved in hepatic lipid handling and energy metabolism were selected from the above pathways and visualized as a log2FC heatmap (Figure 6). Relative to the FO group, *CYP7A1* (a rate-limiting enzyme of bile acid synthesis) was significantly down-regulated in both vegetable-oil groups (padj < 0.05), with the most pronounced decrease in the SO group. Several other genes were significantly altered in only one group: *HSD11B2*, *PLB1* and *DGKB* were down-regulated in the LO group, whereas *CYP17A1* and *PLA2G4C* were down-regulated in the SO group. Among lipid droplet- and trafficking-related genes, *PLIN3* and *APOF* were up-regulated in the LO group, while the lipolysis inhibitor *G0S2* was up-regulated in both groups.

GO enrichment analysis of the DEGs is provided in Appendix A (Figure A1). The significantly enriched terms were related mainly to membrane components, transport, and signal transduction rather than to canonical lipid metabolism categories.

### 3.8. Validation of Key Lipid Metabolism Genes by RT-qPCR

**Figure 7:**
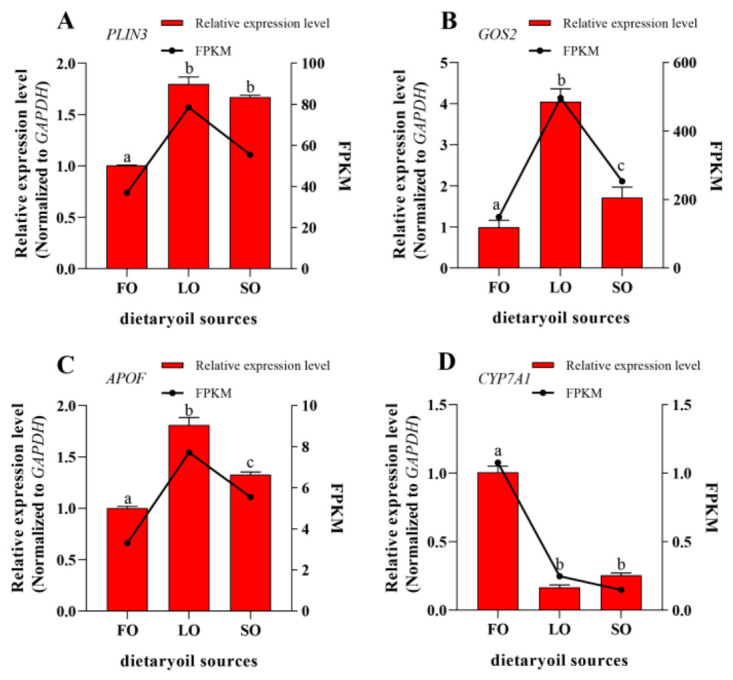
RT-qPCR validation of key hepatic lipid metabolism genes in juvenile *P. sinensis* fed FO, LO and SO diets. Relative expression levels of (**A**) *PLIN3*, (**B**) *G0S2*, (**C**) *APOF*, and (**D**) *CYP7A1*. Expression was normalized to *GAPDH* and calculated using the 2^−ΔΔCq^ method. Values are expressed as mean ± SEM (*n* = 3 pooled biological replicates per group). Bars labeled with different letters differ significantly (*p* < 0.05, Tukey’s HSD test).

To cross-validate the RNA-seq data on an independent platform, four DEGs spanning distinct functional nodes of hepatic lipid handling—lipid-droplet coating, lipolysis suppression, lipid trafficking, and bile-acid synthesis (*PLIN3*, *G0S2*, *APOF*, *CYP7A1*)—were assayed by RT-qPCR (Figure 7). The remaining genes shown in Figure 6 were derived from the same DESeq2 analysis and are therefore reported as RNA-seq-based findings without independent qPCR confirmation. Compared with the FO group, transcript levels of *PLIN3* (Figure 7A), *G0S2* (Figure 7B) and *APOF* (Figure 7C) were significantly elevated in both the LO and SO groups (*p* < 0.05). *CYP7A1* was significantly down-regulated in the LO and SO groups (*p* < 0.05) (Figure 7D). For all four genes, the direction of expression change measured by RT-qPCR was consistent with the corresponding FPKM values from RNA-seq.

## 4. Discussion

### 4.1. Growth Performance and Proximate Composition

The nutritional effects of various vegetable and animal oils have been widely investigated in aquatic animals; however, their application as fish oil substitutes in *P. sinensis* aquaculture remains poorly documented. In the present study, full replacement of the supplemental FO (the 4% added oil) with LO or SO did not compromise growth performance, FCR, or SGR of juvenile *P. sinensis* during the 8-week feeding trial, while the fish meal-derived lipid—the major contributor to total dietary lipid—remained identical across all diets. These findings contrast with observations in swimming crab (*Portunus trituberculatus*), where replacement of FO with LO for 42 days led to reduced WGR and feed efficiency [38]. Similarly, in turbot (*Scophthalmus maximus*), LO substitution for nearly 2 months did not affect weight gain but significantly decreased feed efficiency [39]. In contrast, in the large yellow croaker, full substitution of FO with SO for 12 weeks markedly reduced weight gain rate and SGR [40]. However, those studies generally involved more extensive substitution of the dietary lipid supply, whereas the present design replaced only the supplemental oil fraction and retained the fish meal-derived lipid. The milder dietary perturbation here may help explain why growth was maintained, consistent with the general view that the physiological impact of fish oil replacement scales with the extent of substitution. This suggests a relatively good tolerance of juvenile *P. sinensis* to substitution of the supplemental lipid source, possibly because the retained fish meal-derived lipid sustained a basal supply of n-3 LC-PUFA across all diets; nonetheless, this interpretation requires confirmation over longer feeding periods and across developmental stages.

Consistent with the unaffected growth, full replacement of supplemental FO with LO or SO did not alter crude protein or crude lipid contents in either liver or muscle tissues of juvenile *P. sinensis*. Although previous studies have reported that full replacement of dietary FO with vegetable oils significantly altered hepatic crude lipid content in some teleost species, such as the large yellow croaker [41], tilapia [42], and groupers (*Epinephelus coioides*) [21], the stability of proximate composition in *P. sinensis* suggests that, within the conditions of this study, substituting the supplemental oil source primarily reshaped the quality (fatty acid profile) rather than the quantity of deposited lipid, an aspect examined in detail below.

### 4.2. Tissue Fatty Acid Composition and Its Nutritional Implications

Although growth and proximate composition were unaffected, replacement of the supplemental FO with LO or SO substantially reshaped the fatty acid profiles of both liver and muscle, indicating that the main impact of the substitution was on lipid quality rather than quantity. Both vegetable oil diets markedly increased the relative proportions of LA and total n-6 PUFA, while ALA was elevated specifically in the LO group, mirroring the characteristic fatty acid compositions of the two dietary oils. Concurrently, n-3 LC-PUFA were reduced—EPA decreased significantly in the SO group in the liver and in both groups in muscle, and DHA declined in both tissues—resulting in a significantly lower n-3/n-6 PUFA ratio in the LO and SO groups.

These shifts are consistent with extensive evidence that replacing FO with terrestrial plant oils, which are rich in C18 PUFA but devoid of preformed LC-PUFA, lowers tissue EPA and DHA and disturbs the n-3/n-6 balance [8,43,44]. Notably, the limited retention of EPA and DHA despite an increased dietary supply of their C18 precursors (ALA in LO; LA in SO) suggests that juvenile *P. sinensis* has only a limited capacity to convert C18 PUFA into n-3 LC-PUFA. Mechanistically, LC-PUFA biosynthesis proceeds through sequential desaturation and elongation of C18 precursors, catalyzed mainly by fatty acyl desaturases (e.g., *Fads2*) and elongases (e.g., *Elovl2/5*) [45]. In the present transcriptome, genes encoding these enzymes were expressed but were not differentially expressed among groups, indicating that their transcription was not up-regulated to compensate for the reduced dietary LC-PUFA supply. This lack of compensatory induction, together with the accumulation of C18 precursors and the marked decline in tissue EPA and DHA, is consistent with a constrained desaturation–elongation pathway in *P. sinensis*, as reported in several other freshwater species [46,47]. Collectively, these observations indicate that *P. sinensis* depends substantially on a preformed dietary supply of EPA and DHA to maintain tissue LC-PUFA levels.

From an applied perspective, these compositional changes have implications beyond the animal itself. Because *P. sinensis* is consumed as a food product valued partly for its nutritional quality, the reduction in muscle EPA and DHA (each by approximately 40%) under full substitution of the supplemental oil would lower the n-3 LC-PUFA available to consumers and reduce the n-3/n-6 ratio of the edible tissue—both widely regarded as indicators of the nutritional value of aquatic foods. This represents a key trade-off: although growth and feed efficiency were maintained, the nutritional quality of the edible tissue may be compromised. Partial rather than full substitution, oil blending, or a fish oil finishing phase before harvest may help reconcile reduced FO dependence with preservation of the product’s n-3 LC-PUFA value, and warrants targeted evaluation in *P. sinensis*.

### 4.3. Hepatic Histology and Lipid Deposition

Previous studies have reported that long-term or high-level intake of LO and SO has also been associated with abnormal lipid deposition, even in the absence of growth impairment, potentially affecting hepatic lipid metabolism and storage patterns in aquatic animals. In species including Nile tilapia and the large yellow croaker, increased lipid droplet accumulation and hepatocellular vacuolation have been reported following dietary FO replacement with vegetable oils, indicating altered lipid storage and utilization pathways [5,19]. In the present study, however, neither Oil Red O quantification nor hepatic triglyceride and cholesterol contents differed significantly among the FO, LO and SO groups, and histological examination revealed no appreciable group differences in hepatic vacuolation. The absence of significant changes at the tissue level indicates that, within the 8-week experimental period, full replacement of the supplemental FO with LO or SO did not produce overt hepatic lipid accumulation or dysfunction. Consistently, serum biochemical parameters remained unchanged, supporting the interpretation that any hepatic effects of replacement of supplemental FO were limited over this timeframe.

### 4.4. Hepatic Oxidative Status

Lipid peroxidation is widely regarded as a sensitive indicator of oxidative stress and metabolic imbalance induced by dietary lipid manipulation [48], with MDA widely employed as a biomarker for oxidative damage in aquatic animals fed plant oil-based diets [20]. In this study, full replacement of the supplemental FO with SO significantly elevated hepatic MDA levels, whereas no significant change was observed in the LO group, suggesting oil-specific differences in oxidative stability.

Previous studies in freshwater fish have also shown that high dietary levels of n-6 PUFA derived from SO enhance susceptibility to lipid peroxidation and oxidative stress, which may be attributed to the elevated peroxidation potential of LA-rich membranes [49]. More noteworthy is the absence of a significant rise in MDA in the LO group, which at first sight appears counterintuitive: ALA enriched by the LO diet is more unsaturated than LA and therefore carries a higher theoretical peroxidation index on a per-molecule basis [50]. Several non-exclusive factors may contribute to this apparent discrepancy.

Hepatic ALA accounted for approximately 3.82% of total fatty acids in the LO group, whereas hepatic LA reached about 17.57% in the SO group. Given this substantial difference in the abundance of the readily oxidizable C18 substrate, the larger n-6 pool in the SO liver may provide a larger substrate pool for lipid than the comparatively smaller n-3 pool in the LO liver, despite the higher per-molecule peroxidation index of ALA. This may help explain why hepatic MDA increased significantly in the SO group but not in the LO group.

In addition, no significant changes in antioxidant enzyme activities or in the expression of *SOD1*, *SOD2* and *CAT* were detected, which further suggests that the oxidative stress observed in the SO group represented an early biochemical response rather than a fully developed oxidative imbalance. Within the 8-week period, antioxidant enzyme activities and related gene expression remained unchanged, indicating that the antioxidant defense system was not overwhelmed in any group, consistent with the absence of a significant MDA increase in the LO group. Whether the higher unsaturation of ALA translates into greater peroxidation over longer feeding periods remains to be determined.

### 4.5. Transcriptional Regulation of Hepatic Lipid Metabolism

Hepatic transcriptomic analysis combined with RT-qPCR validation further elucidated the transcriptional responses of lipid metabolism to dietary FO replacement in *P. sinensis*. Whereas previous work on FO replacement in *P. sinensis* has focused mainly on growth performance and tissue fatty acid composition [23,24], to our knowledge the present study is the first to characterize the hepatic transcriptional response to supplemental FO replacement in this species, thereby extending evidence on vegetable oil substitution from teleosts to a cultured reptile.

Although overall growth performance was not significantly compromised, gene expression profiles indicated that LO and SO intake triggered adaptive adjustments in hepatic lipid metabolism. Both heatmap analysis and RT-qPCR validation demonstrated significant down-regulation of *CYP7A1* expression in the LO and SO groups relative to the FO group. As *CYP7A1* is a rate-limiting enzyme for bile acid biosynthesis, its down-regulation may indicate a reduced transcriptional drive for hepatic cholesterol catabolism [51]. Mechanistically, *CYP7A1* governs the classical pathway of bile acid synthesis, the principal route through which hepatic cholesterol is converted to bile acids and removed from the liver; its transcription is regulated by bile acid– and oxysterol-responsive nuclear receptors such as FXR and LXR [52]. In the present transcriptome, FXR and LXR were expressed but not significantly altered among groups, indicating that the *CYP7A1* change was not accompanied by a detectable transcriptional shift in these upstream regulators; the diet-induced change in *CYP7A1* may therefore involve post-transcriptional or substrate-flux-related regulation rather than a pronounced change in receptor transcription. However, hepatic and serum total cholesterol did not differ significantly among groups, so this transcriptional change was not accompanied by detectable alterations in cholesterol content. Therefore, we interpret it as a transcriptional-level adjustment rather than direct evidence of altered cholesterol homeostasis, which would require measurement of bile acids and related metabolites.

Among lipid droplet- and trafficking-related genes, the lipolysis inhibitor *G0S2* was significantly up-regulated in both vegetable oil groups, whereas *PLIN3* and *APOF* reached significance mainly in the LO group by RNA-seq. RT-qPCR confirmed significant up-regulation of all three genes in both groups; the somewhat broader significance detected by RT-qPCR is consistent with its higher sensitivity, and importantly, the direction of change was concordant across both platforms. Up-regulation of *PLIN3*, a protein associated with lipid droplets, is commonly linked to increased intracellular lipid droplet accumulation [53], while *G0S2* is known to suppress lipolysis [54]. Together, these changes provide a plausible molecular explanation for the subtle shift in hepatic lipid handling toward storage-related processes in the LO and SO groups.

Notably, KEGG pathway enrichment analysis revealed no metabolic pathways with significant alterations after multiple-testing correction. This indicates that full replacement of supplemental FO with LO or SO does not appear to fundamentally restructure hepatic lipid metabolic pathways but rather is associated with an altered expression of a limited set of individual genes. Accordingly, we interpret these findings as fine-scale transcriptional adjustments rather than a significant remodeling of hepatic lipid metabolism, consistent with the absence of significantly enriched pathways after multiple-testing correction. Gene Ontology enrichment likewise did not converge on canonical lipid metabolism categories, with significant terms relating mainly to membrane components, transport, and signal transduction; the lack of strong functional convergence on lipid-metabolic pathways in both KEGG and GO analyses further supports this interpretation. Such limited transcriptional changes may help maintain growth performance while modestly adjusting hepatic lipid handling in response to dietary fatty acid composition [55].

### 4.6. Limitations

Several limitations of the present study should be acknowledged when interpreting these findings:

- First, the trial lasted 8 weeks and used juvenile animals, so the results reflect this particular period and life stage and may not extend to longer-term feeding or to other developmental stages. Second, the supplemental FO was replaced entirely by a single vegetable oil without intermediate levels, so dose-dependent relationships and a possible optimal replacement level were not addressed and warrant a graded-substitution study.
- Second, only the 4% supplemental oil was replaced while the fish meal-derived lipid was retained, so total dietary n-3 LC-PUFA could not be reduced to zero; this design constrains the assessment of dose-dependent responses. Resolving dose–response relationships and an optimal replacement level would require eliminating the background fish-derived lipid—for example, by using low-lipid (defatted) fish meal or alternative protein sources—before applying graded levels of oil substitution.
- Third, each biological replicate consisted of RNA pooled from three turtles (*n* = 3 per group); while this approach provided representative group-level expression profiles, analyzing individuals separately in future work would allow inter-individual variability to be assessed. *GAPDH* was used as the sole reference gene for RT-qPCR; although it showed stable Cq values across samples and has been widely applied in *P. sinensis*, future studies would benefit from validating multiple candidate reference genes using tools such as geNorm or NormFinder.
- Fourth, the present analysis focused on the transcriptional level; complementary measurements at the protein, enzyme, and metabolite levels would help confirm the functional relevance of these gene-expression changes.
- Finally, tissue fatty acids were quantified by peak-area normalization and are expressed as relative percentages of total fatty acids rather than absolute concentrations; relative and absolute changes need not coincide, and this should be considered when comparing with studies reporting absolute contents.
- Building on these results, future studies incorporating graded replacement levels, longer feeding periods, individually analyzed samples, and multi-level validation would help to define practical inclusion levels of vegetable oils in *P. sinensis* feeds.

## 5. Conclusions

In conclusion, full replacement of the supplemental dietary fish oil with soybean oil or linseed oil for 8 weeks did not affect growth performance, tissue proximate composition, or serum biochemical parameters in juvenile *P. sinensis*. However, replacement of the supplemental fish oil substantially altered the fatty acid composition of liver and muscle, reduced tissue n-3 LC-PUFA levels, and was associated with elevated hepatic MDA in the soybean oil group. At the transcriptional level, it was associated with down-regulation of *CYP7A1* and up-regulation of *PLIN3*, *G0S2* and *APOF* in the liver, suggesting modest, gene-level adjustments in hepatic cholesterol turnover and lipid storage rather than a global restructuring of hepatic lipid metabolism; these transcriptional observations await confirmation at the protein, enzyme, or metabolite level. Overall, these results indicate that, within an 8-week period, juvenile *P. sinensis* tolerated full replacement of the supplemental fish oil without overt impairment of growth or hepatic health, while showing altered tissue fatty acid profiles—most notably a marked reduction in n-3 LC-PUFA in the edible tissue—and modest changes in the hepatic expression of lipid metabolism genes. The reduction in n-3 LC-PUFA represents a trade-off relevant to the nutritional quality of the product, and strategies such as partial substitution or a fish oil finishing phase before harvest merit evaluation in this context. Given the short duration, the use of juveniles only, and the transcriptional nature of the molecular evidence, these conclusions should be regarded as preliminary, and longer-term studies with functional (protein and metabolite) validation are warranted.

## Figures and Tables

**Table 1:** Details of experimental diet formulation and proximate contents (% dry matter).

Ingredient	FO	LO	SO
White fish meal ^a^	52.00	52.00	52.00
Poultry by-product meal ^a^	6.00	6.00	6.00
Soybean protein concentrate ^a^	10.00	10.00	10.00
α-Starch	22.00	22.00	22.00
Fish oil	4.00	0.00	0.00
Linseed oil	0.00	4.00	0.00
Soybean oil	0.00	0.00	4.00
Microcrystalline cellulose ^b^	1.86	1.86	1.86
Vitamin and mineral Premix ^c^	0.80	0.80	0.80
Ca(H_2_PO_4_)_2_	2.90	2.90	2.90
Cr_2_O_3_	0.10	0.10	0.10
Antioxidant	0.01	0.01	0.01
Mold inhibitor	0.03	0.03	0.03
Choline chloride	0.20	0.20	0.20
Phagostimulant ^d^	0.10	0.10	0.10
Total	100	100	100
Proximate contents (% dry matter)			
Crude protein	45.15	45.12	45.03
Crude lipid	7.84	7.91	7.78

^a^ Measured crude protein (CP) and crude lipid (CL) contents of the protein ingredients: white fish meal, 65.62% CP and 5.92% CL; poultry by-product meal, 65.92% CP and 11.21% CL; soybean protein concentrate, 64.78% CP and 1.42% CL. ^b^ Microcrystalline cellulose: an inert, non-nutritive filler. ^c^ Vitamin and mineral Premix according to Dai et al. [26]. ^d^ Phagostimulant: Betaine.

**Table 2:** Fatty acid composition of the experimental diet.

Fatty Acid (%)	FO	LO	SO
C14:0	3.33 ± 0.02	3.64 ± 0.12	3.20 ± 0.09
C16:0	19.55 ± 0.44 ^a^	16.72 ± 0.00 ^b^	17.00 ± 0.02 ^b^
C18:0	4.30 ± 0.04 ^a^	4.20 ± 0.04 ^a^	3.37 ± 0.05 ^b^
SFA	27.17 ± 0.49 ^a^	24.55 ± 0.15 ^b^	23.56 ± 0.06 ^b^
C16:1n-7	3.92 ± 0.06	4.07 ± 0.04	4.08 ± 0.02
C18:1n-9	19.29 ± 0.03 ^a^	20.18 ± 0.05 ^b^	19.40 ± 0.07 ^a^
MUFA	23.21 ± 0.09 ^a^	24.25 ± 0.09 ^b^	23.47 ± 0.08 ^a^
C18:2n-6(LA)	24.80 ± 0.63 ^a^	28.05 ± 0.07 ^b^	33.17 ± 0.06 ^c^
C20:4n-6	0.44 ± 0.01	0.45 ± 0.01	0.43 ± 0.01
C22:4n-6	0.78 ± 0.01	0.62 ± 0.01	0.62 ± 0.01
n-6 PUFA	26.01 ± 0.62 ^a^	29.12 ± 0.07 ^b^	34.21 ± 0.06 ^c^
C18:3n-3(ALA)	5.17 ± 0.06 ^a^	8.21 ± 0.02 ^b^	5.02 ± 0.04 ^a^
C20:5n-3(EPA)	8.12 ± 0.00 ^a^	6.12 ± 0.00 ^b^	6.03 ± 0.02 ^b^
C22:5n-3	0.70 ± 0.01	0.72 ± 0.01	0.67 ± 0.02
C22:6n-3(DHA)	7.68 ± 0.03 ^a^	5.01 ± 0.03 ^b^	5.03 ± 0.01 ^b^
n-3 PUFA	21.67 ± 0.02 ^a^	20.05 ± 0.02 ^b^	16.74 ± 0.05 ^c^
n-3/n-6PUFA	0.83 ± 0.02 ^a^	0.69 ± 0.00 ^b^	0.49 ± 0.00 ^c^

Data represent the mean ± SEM of two replicate analyses. Within a row, different superscripts exhibit significant differences (*p* < 0.05, Tukey’s test). Several minor fatty acids—C14:1, C17:1n-7, C20:1n-9, C20:2n-6, C20:3n-6, C22:0, C22:1n-11, C22:5n-3 and C24:0—were present only at trace levels and were therefore omitted from the table.

**Table 3:** Sequences of primers used in RT-qPCR.

Gene	Forward Primer (5′–3′)	Reverse Primer (5′–3′)	Accession No.	Efficiency (%)	R^2^
*GAPDH*	GGCTTTCCGTGTTCCAACTC	GACAACCTGGTCCTCCGTGTATC	XM_075920840.1	99.3	0.9997
*CAT*	TTCTCCACTGTTGCTGGGGA	GAAGATGGGAGTGTTGTTGCC	NM_001286934.1	107.4	0.9960
*SOD1*	AGGGCGTCATCAACTTCGAG	ACCTGCACTGGTACATCCATT	XM_006126060.4	104.0	0.9994
*SOD2*	AAGTTCAATGGTGGGGGTCA	GGCTTCCATCAATTCCCCTTG	NM_001317049.1	103.1	0.9961
*PLIN3*	GAGCAGGAGTGAGAGTTTCCC	GGAGACCATGTCGTAGGCAG	XM_075903053.1	90.5	0.9980
*G0S2*	GGGAAAAGCAAAGCGAAGGG	GAGTCTGCCTCTGGAAAGCC	XM_075910087.1	95.4	0.9986
*APOF*	CAGCATTCAAAGTGGGCGT	CTCAGGTTTATTCACACTCGCC	XM_075901870.1	97.1	0.9989
*CYP7A1*	CTATCCTGACCCCTTGACATTC	TGTGACCGTTGCGATAGAAG	XM_075920658.1	105.4	0.9953

*GAPDH*: glyceraldehyde-3-phosphate dehydrogenase; *CAT*: catalase; *SOD1*: superoxide dismutase 1; *SOD2*: superoxide dismutase 2; *PLIN3*: perilipin-3; *G0S2*: G0/G1 switch 2; *APOF*: apolipoprotein F; *CYP7A1*: cytochrome P450 family 7 subfamily A member 1.

**Table 4:** Survival rate and growth performance of juvenile *P. sinensis* fed the experimental diets.

Parameters	FO	LO	SO
Initial body weight (IBW, g)	55.03 ± 0.05	54.95 ± 0.10	54.97 ± 0.12
Final body weight (FBW, g)	153.28 ± 1.87	148.56 ± 0.37	151.99 ± 0.97
SR (%)	83.33 ± 3.33	78.89 ± 6.76	80.00 ± 6.94
WGR (%)	178.54 ± 3.40	170.35 ± 0.67	176.51 ± 1.77
SGR (% d^−1^)	1.83 ± 0.02	1.78 ± 0.00	1.82 ± 0.01
FCR	1.15 ± 0.02	1.20 ± 0.05	1.16 ± 0.01
VSI (%)	9.36 ± 0.14	9.40 ± 0.13	9.50 ± 0.08
HSI (%)	4.48 ± 0.05	4.60 ± 0.09	4.72 ± 0.14

Values are mean ± SEM (*n* = 3).

**Table 5:** The contents of crude protein and crude lipid in muscle and liver of juvenile *P. sinensis* fed the experimental diets.

Index (% Wet Weight)	Liver			Muscle		
	FO	LO	SO	FO	LO	SO
Crude protein	16.83 ± 0.22	16.9 ± 0.18	17.16 ± 0.14	14.93 ± 0.07	15.12 ± 0.10	14.83 ± 0.09
Crude lipid	3.75 ± 0.11	3.82 ± 0.13	3.91 ± 0.12	2.29 ± 0.08	2.42 ± 0.10	2.48 ± 0.05

Values are mean ± SEM (*n* = 3).

**Table 6:** Liver and muscle fatty acid composition of juvenile *P. sinensis* fed the experimental diets (% total fatty acid).

Fatty Acid (%)	Liver			Muscle		
	FO	LO	SO	FO	LO	SO
C14:0	3.35 ± 0.27	2.69 ± 0.08	2.86 ± 0.18	1.79 ± 0.23 ^a^	0.96 ± 0.20 ^b^	0.61 ± 0.08 ^b^
C16:0	35.50 ± 0.51	34.02 ± 0.64	35.04 ± 0.47	53.86 ± 0.37 ^a^	48.30 ± 0.08 ^b^	41.20 ± 0.69 ^c^
C18:0	13.42 ± 0.94	13.79 ± 0.27	11.42 ± 0.18	17.24 ± 0.63	21.45 ± 1.39	21.88 ± 1.16
ΣSFA	52.27 ± 0.41 ^a^	50.51 ± 0.83 ^ab^	49.33 ± 0.47 ^b^	72.88 ± 0.68 ^a^	70.71 ± 1.64 ^a^	63.69 ± 1.64 ^b^
C16:1n-7	27.65 ± 1.11 ^a^	22.82 ± 1.51 ^b^	23.07 ± 0.88 ^b^	4.09 ± 0.35 ^a^	3.83 ± 0.65 ^a^	2.05 ± 0.33 ^b^
C18:1n-9	9.87 ± 0.38 ^a^	9.77 ± 0.26 ^a^	6.91 ± 0.16 ^b^	6.65 ± 0.41	7.20 ± 0.24	7.00 ± 0.30
ΣMUFA	37.52 ± 0.73 ^a^	32.60 ± 1.27 ^b^	29.98 ± 0.89 ^b^	10.74 ± 0.68	11.03 ± 0.87	9.05 ± 0.60
C18:2n-6(LA)	2.76 ± 0.39 ^a^	10.32 ± 1.63 ^b^	17.57 ± 1.02 ^c^	2.30 ± 0.27 ^a^	7.41 ± 0.69 ^b^	17.63 ± 0.57 ^c^
C20:4n-6	0.27 ± 0.04 ^a^	0.15 ± 0.03 ^ab^	0.11 ± 0.02 ^b^	0.53 ± 0.27	0.56 ± 0.10	0.99 ± 0.12
n-6 PUFA	3.04 ± 0.36 ^a^	10.46 ± 1.64 ^b^	17.96 ± 1.04 ^c^	2.83 ± 0.33 ^a^	7.97 ± 0.79 ^b^	18.63 ± 0.69 ^c^
C18:3n-3(ALA)	1.78 ± 0.08 ^a^	3.82 ± 0.12 ^b^	1.14 ± 0.03 ^c^	1.21 ± 0.04 ^a^	2.43 ± 0.08 ^b^	1.06 ± 0.09 ^a^
C20:5n-3(EPA)	1.23 ± 0.13 ^a^	0.94 ± 0.17 ^a^	0.34 ± 0.09 ^b^	5.61 ± 0.68 ^a^	3.08 ± 0.15 ^b^	3.32 ± 0.29 ^b^
C22:6n-3(DHA)	1.34 ± 0.05 ^a^	0.63 ± 0.07 ^b^	0.35 ± 0.07 ^c^	4.43 ± 0.17 ^a^	2.44 ± 0.41 ^b^	2.75 ± 0.16 ^b^
n-3 PUFA	4.35 ± 0.14 ^a^	5.40 ± 0.28 ^b^	1.83 ± 0.13 ^c^	11.24 ± 0.71 ^a^	7.94 ± 0.48 ^b^	7.13 ± 0.37 ^b^
n-3/n-6 PUFA	1.43 ± 0.18 ^a^	0.52 ± 0.09 ^b^	0.10 ± 0.00 ^b^	4.13 ± 0.67 ^a^	1.00 ± 0.05 ^b^	0.38 ± 0.01 ^b^

Values are mean ± SEM (*n* = 3). Within a row, different superscripts exhibit significant differences (*p* < 0.05, Tukey’s test). Several minor fatty acids—C14:1, C17:1n-7, C20:1n-9, C20:2n-6, C20:3n-6, C22:0, C22:1n-11, C22:5n-3 and C24:0—were present only at trace levels and were therefore omitted from the table.

**Table 7:** The serum biochemical parameters of juvenile *P. sinensis* fed the experimental diets.

Parameters	FO	LO	SO
TG (mmol/L)	4.59 ± 0.59	4.20 ± 0.68	4.55 ± 0.72
T-CHO (mmol/L)	7.50 ± 0.21	7.25 ± 0.24	7.44 ± 0.30
HDL-C (mmol/L)	3.50 ± 0.13	4.06 ± 0.35	3.74 ± 0.33
LDL-C (mmol/L)	4.28 ± 0.46	4.33 ± 0.24	4.15 ± 0.37
AST (U/L)	28.99 ± 2.84	30.19 ± 1.82	34.61 ± 2.45
ALT (U/L)	4.12 ± 0.99	3.08 ± 0.73	4.75 ± 1.15

Values are mean ± SEM (*n* = 3). TG, triglyceride; T-CHO, total cholesterol; HDL-C, high-density lipoprotein cholesterol; LDL-C, low-density lipoprotein cholesterol; AST, aspartate aminotransferase; ALT, alanine transaminase.

## Data Availability

The raw RNA-seq sequencing data generated in this study are openly available in Zenodo at https://doi.org/10.5281/zenodo.19660547.

## Appendix A

### Appendix A.1

**Table A1:** Sequencing data quality control summary.

Sample	Raw Reads	Raw Bases	Clean Reads	Clean Bases	Q20 (%)	Q30 (%)	GC (%)	RIN
FO1	45,141,040	6.77 G	44,627,948	6.69 G	98.92	94.93	48.66	6.70
FO2	46,928,836	7.04 G	46,374,474	6.96 G	98.88	94.83	48.48	6.60
FO3	48,366,338	7.25 G	47,781,712	7.17 G	98.77	94.33	48.38	6.70
LO1	47,975,436	7.20 G	47,463,036	7.12 G	98.88	94.84	48.16	5.90
LO2	50,144,368	7.52 G	49,650,334	7.45 G	98.93	95.07	48.08	6.00
LO3	47,169,644	7.08 G	46,674,080	7.00 G	98.92	95.03	48.33	6.10
SO1	50,739,402	7.61 G	50,184,536	7.53 G	98.92	94.95	48.34	7.30
SO2	47,187,690	7.08 G	46,702,152	7.01 G	98.87	94.78	48.01	7.20
SO3	47,607,272	7.14 G	47,092,154	7.06 G	98.9	95.03	48.44	7.40

### Appendix A.2

**Table A2:** Summary of read mapping rates against the reference genome.

Sample	Total Reads	Total Mapped	Unique Mapped	Multi Mapped
FO1	44,627,948	37,668,649 (84.41%)	36,076,758 (80.84%)	1,591,891 (3.57%)
FO2	46,374,474	39,276,153 (84.69%)	37,644,851 (81.18%)	1,631,302 (3.52%)
FO3	47,781,712	40,495,853 (84.75%)	38,844,870 (81.3%)	1,650,983 (3.46%)
LO1	47,463,036	40,407,026 (85.13%)	38,753,287 (81.65%)	1,653,739 (3.48%)
LO2	49,650,334	42,365,845 (85.33%)	40,631,211 (81.83%)	1,734,634 (3.49%)
LO3	46,674,080	39,669,426 (84.99%)	38,026,211 (81.47%)	1,643,215 (3.52%)
SO1	50,184,536	42,576,445 (84.84%)	40,809,974 (81.32%)	1,766,471 (3.52%)
SO2	46,702,152	39,782,867 (85.18%)	38,164,149 (81.72%)	1,618,718 (3.47%)
SO3	47,092,154	39,945,876 (84.82%)	38,292,537 (81.31%)	1,653,339 (3.51%)
